# Pressureless and Low-Pressure Synthesis of Microporous Carbon Spheres Applied to CO_2_ Adsorption

**DOI:** 10.3390/molecules25225328

**Published:** 2020-11-15

**Authors:** Iwona Pełech, Daniel Sibera, Piotr Staciwa, Urszula Narkiewicz, Robert Cormia

**Affiliations:** 1 Department of Chemical and Environment Engineering, Faculty of Chemical Technology and Engineering, West Pomeranian University of Technology in Szczecin, Pułaskiego 10, 70-322 Szczecin, Poland; Iwona.Pelech@zut.edu.pl (I.P.); daniel.sibera@zut.edu.pl (D.S.); piotr.staciwa@zut.edu.pl (P.S.); 2 Faculty of Civil and Environmental Engineering, West Pomeranian University of Technology in Szczecin, al. Piastów 50a, 70-311, 70-322 Szczecin, Poland; 3 Chemistry Faculty, Chemistry Department, Foothill College, 12345 El Monte Road, Los Altos Hills, CA 94022, USA; cormiarobert@fhda.edu

**Keywords:** carbon spheres, carbon nanospheres, carbon dioxide adsorption, autoclave, low-pressure synthesis, pressureless, resorcinol

## Abstract

In this work, low-pressure synthesis of carbon spheres from resorcinol and formaldehyde using an autoclave is presented. The influence of reaction time and process temperature as well as the effect of potassium oxalate, an activator, on the morphology and CO_2_ adsorption properties was studied. The properties of materials produced at pressureless (atmospheric) conditions were compared with those synthesized under higher pressures. The results of this work show that enhanced pressure treatment is not necessary to produce high-quality carbon spheres, and the morphology and porosity of the spheres produced without an activation step at pressureless conditions are not significantly different from those obtained at higher pressures. In addition, CO_2_ uptake was not affected by elevated pressure synthesis. It was also demonstrated that addition of the activator (potassium oxalate) had much more effect on key properties than the applied pressure treatment. The use of potassium oxalate as an activator caused non-uniform size distribution of spherical particles. Simultaneously higher values of surface area and total pore volumes were reached. A pressure treatment of the carbon materials in the autoclave significantly enhanced the CO_2_ uptake at 25 °C, but had no effect on it at 0 °C.

## 1. Introduction

Carbon dioxide concentrations in the atmosphere continue to rise and are currently approaching 420 ppm, coincident with rising global temperatures and climate change (CDIAC). CO_2_ emissions from the use of fossil fuels account for roughly 65% of the global greenhouse gas burden and are estimated to exceed 1 trillion tons CO_2_ as added from industrial activity. The ambitious objective of the European Green Deal is to achieve neutral emissions by 2050 [1] in accordance with United Nations Sustainable Development Goals. To meet this objective, new and efficient technologies for carbon dioxide sequestration and its selective transformation to useful chemical products will be needed at scale [2,3].

Carbon dioxide separation and sequestration can be performed using refrigeration [4], absorption in liquids [5], or adsorption [6] (including membrane separation [7]. To perform CO_2_ adsorption, various porous sorbents can be used, including zeolites, mesoporous silica, metal organic frameworks or carbon materials [8], including activated carbon [9]. Microporous carbon spheres have recently attracted attention due to their facile synthesis, low price, gas adsorption efficiency, and kinetics [10,11].

There are several methods used to produce carbon spheres, including arc discharge [12], laser ablation [13], chemical vapor deposition (CVD) (decomposition of chemical substances containing carbon), and pyrolysis and catalytic decomposition of organic compounds by heat treatment of polymers or other materials, often in an autoclave [14,15,16]. In the latter approach, reactants are placed in various types of metal autoclaves and usually heated to a (rather low) desired temperature. Recently, microwave reactors have attracted more attention as a new efficient heating method [17,18]. Several parameters, e.g., carbon source, temperature, pH, or reaction time, can influence the morphology and properties of carbon spheres. Carbon sources including phenol [19,20] or resorcinol–formaldehyde resin [21,22,23,24] can be used. During the preparation of carbon spheres from resorcinol–formaldehyde resin, potassium oxalate [24], potassium hydroxide [25,26,27], and potassium carbonate [28] can be used as activating agents. These highly alkaline chemical agents support development of pore structure through dehydration and polymeric structure degradation [29]. Wang et al. [30] successfully used ZnCl_2_ as the activating agent.

Many authors, including Wickramaratne and Jaroniec, have underlined the importance of small pores for efficient carbon dioxide adsorption [27] which was confirmed by Lee et al. [31]. Casso et al. [32] asserted that gas adsorption in pores depended on the applied pressure and the increase in adsorption pressure, with adsorption being governed by larger pores. Nonetheless, researchers claim that at atmospheric pressure, adsorption in narrow micropores (below 0.6 nm) enhance adsorption. Studies by Chen et. al. [33] demonstrated that adsorption of CO_2_ molecules in 0.3 nm slit pores (slightly smaller than the CO_2_ kinetic diameter), was very poor. By contrast, significant CO_2_ adsorption was observed in 0.4 nm pores. Additionally, for pores larger than 0.4 nm, a significant decrease in stabilization energy of CO_2_ molecules was observed. With an increase in pore size, interactions between CO_2_ molecules and carbon pores become weaker.

Taking into account the importance of micropores for efficient carbon dioxide adsorption, we produced carbon spheres with high micropore size distribution. In our previous paper, we investigated the influence of different experimental conditions—e.g., reaction time, pressure, and power—on morphology as well as carbon dioxide adsorption properties [34]. The importance of micropores below 0.4 nm for adsorption of carbon dioxide was also shown in this work. The use of a microwave-assisted solvothermal reactor enabled a significant reduction in reaction time, as low as 10 min, compared with processes carried out in autoclaves, lasting for several hours. It was additionally discovered that higher reactor pressures, over 3 MPa, resulted in the collapse of spherical shape and the formation of typical graphene layers.

The aim of the research described in the present paper was to investigate the opposite effect; i.e., how low-pressure or pressureless (atmospheric) conditions during the synthesis of carbon spheres influences morphology, microporous structure, and CO_2_ adsorption efficiency. The effect of reaction time, temperature, and addition of activating agent during formation of carbon spheres from resorcinol and formaldehyde through synthesis in an autoclave was also investigated and discussed.

## 2. Results and Discussions

**Figure 1:**
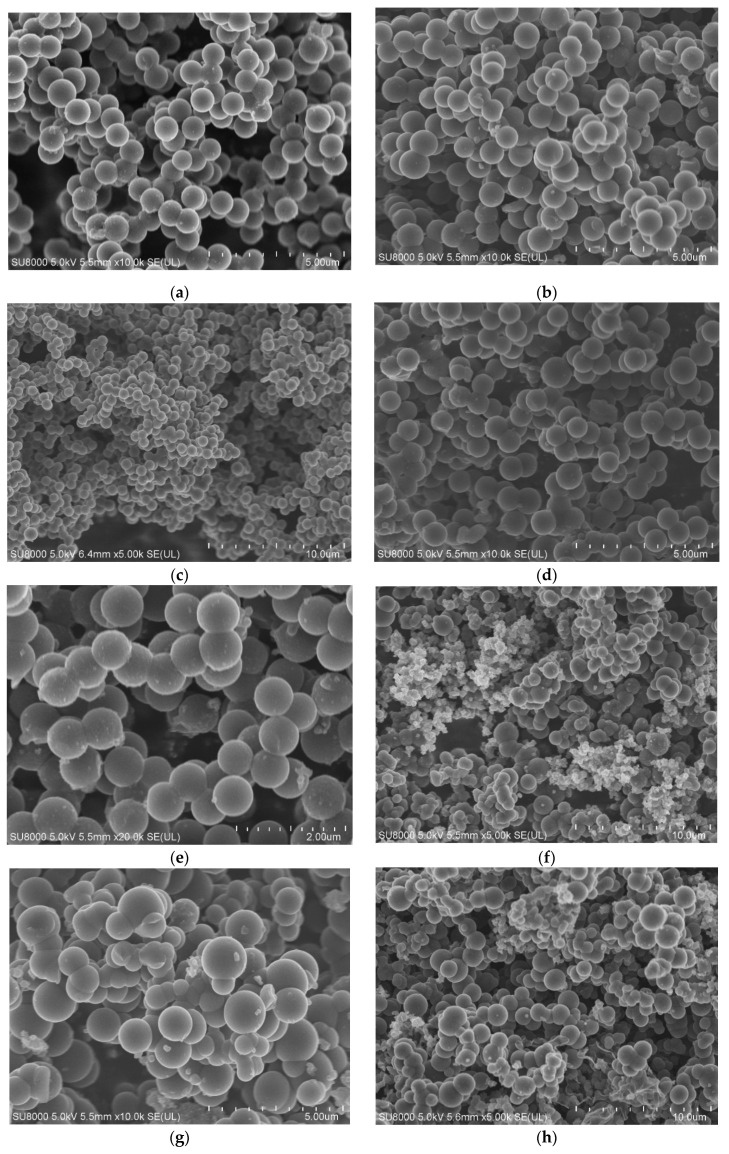

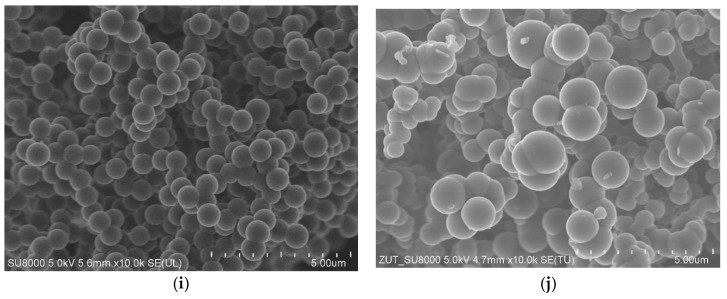
SEM images of the materials obtained under different experimental conditions. (**a**) ARF_15 min/120; (**b**) ARF_ 1 h/120; (**c**) ARF_ 12 h/120; (**d**) ARF_15 min/200; (**e**) ARF_1 h/200; (**f**) ARF_7/1_15 min/120; (**g**) ARF_7/1_1 h/120; (**h**) ARF_7/1_12 h/200; (i) RF; (j) RF_7/1.

Morphology of the samples was investigated using scanning electron microscopy; representative SEM images are presented in Figure 1. Regardless of the process conditions, carbon spheres with relatively uniform diameter were obtained. Figure 1a presents the homogeneous carbon spheres prepared at 120 °C for 15 min with diameters in the range of 700–800 nm. It was found that extending the reaction time to 1 h (Figure 1b) and as high as 12 h (Figure 1c) had no effect on the morphology of carbon spheres or the diameter. Comparing samples ARF_15 min/120 (Figure 1a) and ARF_15 min/200 (Figure 1d) as well as ARF_ 1 h/120 (Figure 1b) and ARF_1 h/200 (Figure 1e) showed that, like reaction time, increasing reaction temperature did not significantly change the morphology or the diameter of the spheres.

Materials produced with potassium oxalate as an activator are presented in Figure 1f–h and were significantly different from those obtained without the addition of potassium salt, especially with respect to their size distribution. Even under mild conditions, 15 min and 120 °C (Figure 1f), in spite of carbon spheres with diameters in the range of 1000–1200 nm, the smaller structures were visible. Additionally, some inclusions were observed and suggest incomplete reaction of potassium oxalate during the preparation procedure. Some inclusions also appeared in the sample obtained at a higher temperature, ARF_7/1_12 h/200 (Figure 1h), as shown in Figure 1h (bottom right corner).

Our studies confirm that pressure treatment is not necessary to produce carbon spheres. Figure 1i,j present materials prepared with and without addition of potassium oxalate, respectively. It is clearly visible that material denoted as RF is the most homogenous of all. Carbon spheres with a mean diameter of about 700 nm with regular spherical shapes and smooth surface were observed, with no inclusions or impurities. Contrary to this, the sample denoted as RF_7/1 exhibited smaller carbon spheres with diameters of about 200 nm, as well as larger spheres with diameters in the range of 1200–1400 nm, similar or even larger like in the case of the samples treated in the autoclave. The results show the presence of non-uniform-sized spherical particles in the sample, probably due to the presence of unreacted potassium oxalate rather than the experimental conditions (temperature or the reaction time). Similar results were described by Ludwinowicz and Jaroniec [24]. Those authors indicated that carbon spheres prepared without salt addition were more uniform, whereas the activated spheres had somewhat irregular shapes and larger diameters. In our case, carbon spheres had larger diameters as well, but the main feature observed was their non-homogenous character. When activating agent in the form of potassium oxalate was added, despite the larger spheres, smaller spherical balls appeared as well. Xu et al. [35] also observed similar behavior, although they prepared carbon microspheres in a hydrothermal reaction of saccharide solutions in the presence of potassium hydroxide.

For the samples with addition of potassium oxalate, a larger degree of merging between spheres was also observed (Figure 1e,g). Rey-Raap et al. [17] also observed that the activation process of carbon spheres leads to a larger degree of merging between spheres and claimed that it can be associated with the presence of oxygenate functional groups and their strong interactions with CO_2_ at high temperatures.

**Figure 2:**
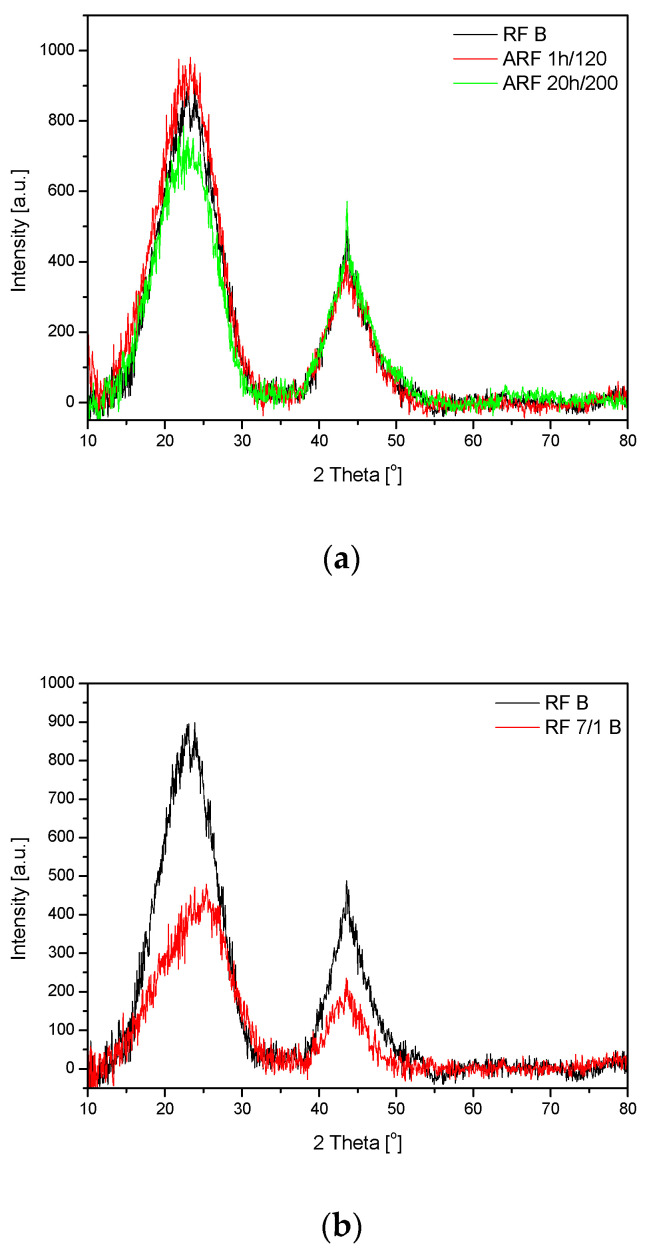
X-ray diffraction patterns of the samples without addition of potassium oxalate and treated under different experimental conditions (**a**) and the samples not treated in the autoclave (**b**).

The phase composition of the samples was examined using XRD (Figure 2). In Figure 2a, the diffraction patterns of the samples obtained without addition of potassium oxalate are presented. It was observed that the varied synthesis conditions had no significant effect on the phase composition of the obtained materials, regardless of whether the samples were treated in an autoclave or not. XRD patterns showed two strong peaks: the first at around 24° and second at around 44°, which can be assigned to the stacking carbon layer structure (002) and ordered graphitic carbon structure (100) [36]. Broadening of the two peaks suggests a low degree of graphitization and the possible presence of amorphous carbon [37,38]. For the sample denoted as ARF_20 h/200 (treated under higher temperature (200 °C) for a longer time (20 h), a decrease of 002 peak intensity and increase of 100 peak intensity was observed in comparison with the samples prepared under mild conditions (ARF_1 h/120) or with the sample produced under pressureless conditions (RF). This indicates an increase in the degree of graphitization. Additionally, a sharper 100 peak with greater intensity was detected for the material denoted as ARF_20 h/200, which can suggest the presence of a crystalline carbon phase within the carbon spheres [39]. These observations correlate well with the results obtained using scanning electron microscopy (SEM), where the formation of graphitic layers under higher temperature has been observed (Figure 1h).

For the samples with addition of potassium oxalate (the diffraction patterns not shown here), similar results as described above were also obtained. 

Comparing the XRD patterns of the samples obtained under the same conditions but with (RF_7/1) or without (RF) potassium oxalate (Figure 2b), it was found that both the intensity of 002 peak and 100 peak for the activated material decreased. This behavior was observed for all the samples regardless of the process conditions. It can indicate that the graphitization degree was reduced in the presence of the activator, which is consistent with the results obtained using scanning electron microscopy.

**Figure 3:**
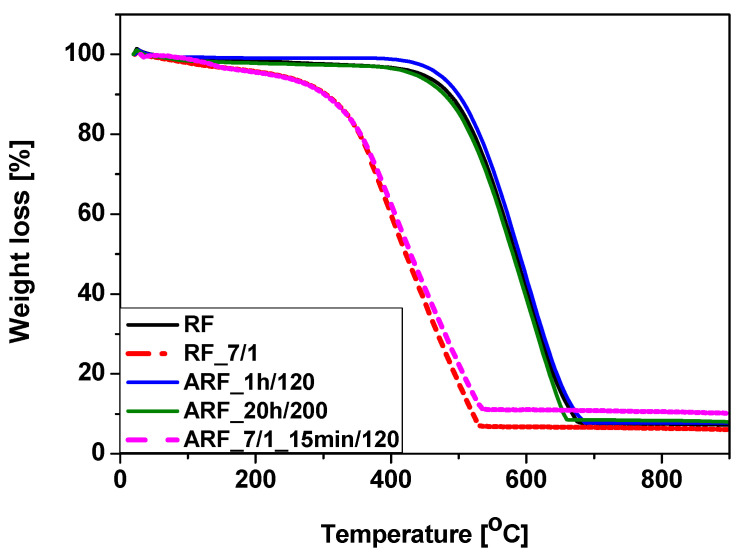
Thermogravimetric curves of the tested samples.

Thermal stability of the samples was studied using a thermogravimetric method, and the thermogravimetric curves are presented in Figure 3. It is clearly visible that the materials activated with potassium oxalate (RF_7/1 and ARF_7/1_15 min/120) have a lower initial combustion temperature than unmodified samples (RF, ARF_1 h/120, and ARF_20 h/200). This can be related to the presence of oxygen functional groups on the carbon spheres surface derived from potassium oxalate and the presence of the amorphous carbon [40]. The lower thermal stability of modified oxalate potassium samples may also result from the fact that, during synthesis, potassium hollows out carbon spheres, creating additional channels with pores. Arising from additional channels on the surface of the spheres, oxygen has easier access to a larger surface of the sample during the thermogravimetric test, which could be the reason for combustion at lower temperatures.

Simultaneously, the final combustion temperature for the activated samples was about 535 °C, in contrast to 680 °C for the non-activated materials. It is known that the carbon structures with a better crystalline structure oxidize at higher temperatures, i.e., 660–760 °C [41,42].

It needs to be highlighted that the thermal stability of the samples mainly depends on the activation process. At the same time, the fact is that whether samples were subjected to the heat treatment in an autoclave or not had no effect on the thermal stability of the materials. This suggests that the carbon spheres obtained either with or without pressure treatment had the same ordered structure, and the degree of orderliness was correlated with the activation process.

**Figure 4:**
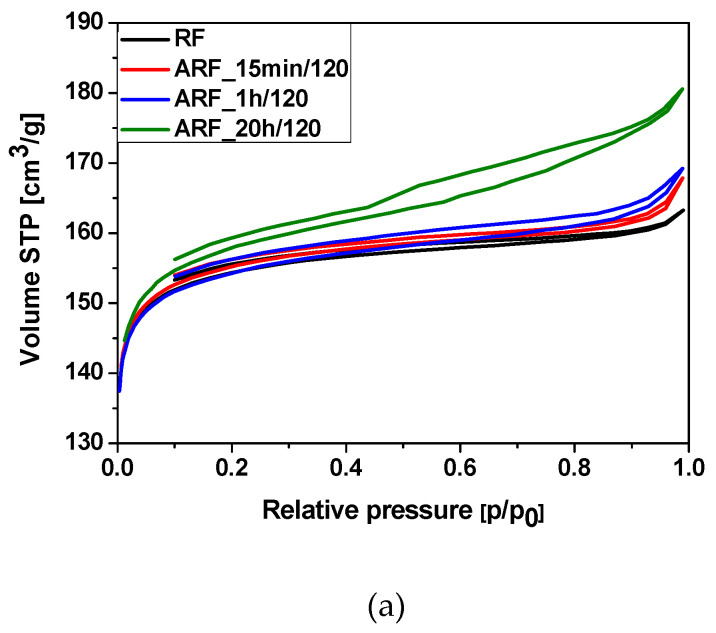

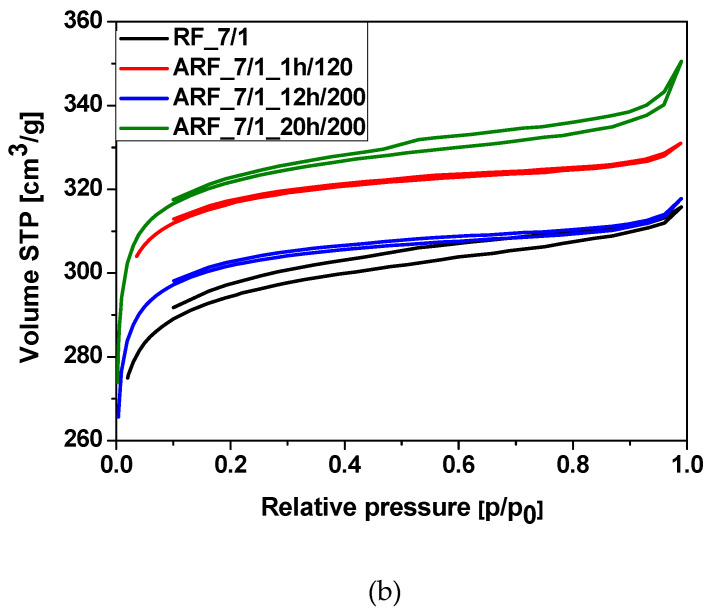
Nitrogen adsorption–desorption isotherms for the samples obtained without (**a**) and with (**b**) addition of potassium oxalate.

Nitrogen adsorption data were used to evaluate the specific surface area and porosity of the obtained materials. The results are shown in Table 1, and low-temperature nitrogen adsorption–desorption isotherms are presented in Figure 4.

The N_2_ adsorption data show these samples have significant macro-micropore structure. Materials produced in this study show mixed I type and II isotherms according to IUPAC classification. Figure 4a shows exemplary nitrogen adsorption isotherms of the unmodified materials. Samples produced without heat treatment adsorbed the lowest amount of nitrogen. With increasing heat treatment time, the amount of the adsorbed nitrogen also increased. Nonetheless, materials obtained using a shorter time for heat treatment show isotherm types more similar to type I, which is characteristic of the microporous materials. With increasing reaction time, the adsorption isotherms were more of type II for the samples in the autoclave for 20 h, suggesting a high proportion of macropores. Increasing reaction time in carbonaceous materials develops the macropores. Type II isotherms also represent unrestricted monolayer-multilayer adsorption. Isotherms similar to those shown here can be assigned to physisorption of N_2_ gases on nonporous or macroporous adsorbents. Interestingly, all samples have an H4 type hysteresis loop. This may indicate the presence of narrow slit pores in the samples. H4 loops are often found in micro-mesoporous carbons [43,44].

Typical nitrogen adsorption isotherms for samples modified with potassium oxalate are shown in Figure 4b. All isotherms are a mix of type I and II, with a high content of micropores. Varying time of heat treatment did not affect the surface of the materials. All of these samples also have an H4 type hysteresis loop.

Compared to unmodified materials, the addition of potassium oxalate resulted in a development of the specific surface area (Table 1). In the samples unmodified with potassium oxalate, the specific surface area was practically constant, between 462 and 486 m^2^/g. In these materials, time and temperature of the process did not affect the value of the specific surface area. Addition of potassium oxalate to the carbon materials during synthesis led to the development of specific surface area, up to 986 m^2^/g (sample ARF_7/1_20 h/200). This is probably due to the carbonization of samples at 700 °C, where potassium ions are considerably intercalated into the carbon spheres to form pores [45,46,47]. The additional pores created in this way increased the specific surface area of the obtained samples and their adsorption capacity. The existence of interconnecting macropores and tunnels could be beneficial for CO_2_ adsorption.

The ratio of the specific surface area to the porosity is similar, both for activated and non-activated samples.

Table 1 shows physicochemical properties of the obtained samples. For the unmodified samples, the average CO_2_ adsorption at 0 and 25 °C was about 3.60 and 2.50 mmol/g, respectively. Additional heat treatment in the autoclave did not improve CO_2_ adsorption values, regardless of reaction time or temperature treatment.

**Figure 5:**
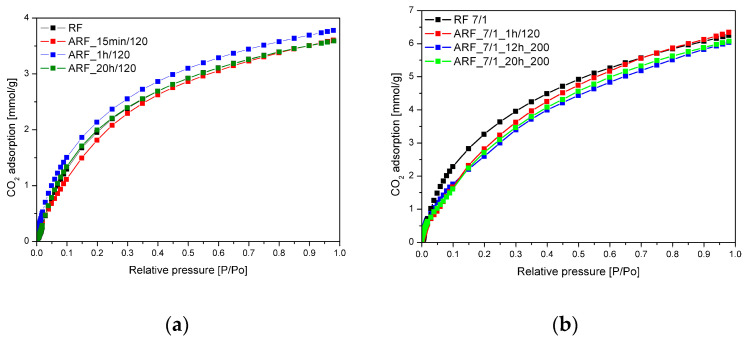
Exemplary CO_2_ adsorption isotherms at 0 °C for the unmodified samples (**a**) and samples modified with potassium oxalate (**b**).

Figure 5 shows the comparison of CO_2_ uptake isotherms at 0 °C for the unmodified samples as well as for the samples modified with potassium oxalate. It is worth noting the strong influence of chemical activation on CO_2_ adsorption. Values for CO_2_ adsorption for samples modified with potassium oxalate were much higher. Activation with potassium ions resulted in development of the surface area and porosity of the carbon materials.

It is noteworthy that the lowest CO_2_ adsorption value at 25 °C was for the untreated sample. Treatment in the autoclave significantly improves CO_2_ adsorption at 25 °C, from 3.74 mmol/g for untreated sample to 4.60 mmol/g for the sample after 1 h of treatment. The values for adsorption at 25 °C were scattered, and no correlation between heat treatment conditions and adsorption values could be observed. Comparing other reported CO_2_ uptake values (Table 2), adsorption on these samples is very good. The high CO_2_ uptake at 25 °C suggests these materials are promising for industrial carbon dioxide capture.

It is generally known that with an increase in temperature, there is a transition from physisorption to chemisorption. In one of our previous papers [48], we described CO_2_ adsorption studies on commercial activated carbon (pure and modified with KOH) using temperature-programmed desorption method (TPD-CO_2_) performed under atmospheric pressure at three different temperatures: −30, 0, and 20 °C. Significant changes in adsorption energy were observed with increasing temperature, corresponding to the growing contribution of chemisorption to physisorption. We concluded that at higher temperatures, the adsorption of carbon dioxide on activated carbon had a mixed (physical/chemical) character and that two types of adsorption sites are present at the surface.

In the case of the present studies, the contribution of chemisorption in relation to physisorption at 25 °C was also higher than at 0 °C.

The influence of porosity of carbon materials on CO_2_ uptake has been widely described [27,31,52]. This is why the relationship between pore size distribution and CO_2_ uptake was also investigated. For CO_2_ adsorption at ambient conditions, ultramicropores below 0.6 nm are highly desirable [32]. In these narrow pores, stronger van der Waals interaction occurs between CO_2_ molecules and pore walls. Due to the average kinetic diameter of the CO_2_ molecule (0.33 nm) [9], the presence of pores between 0.35 and 0.6 nm strongly improves CO_2_ adsorption.

**Figure 6:**
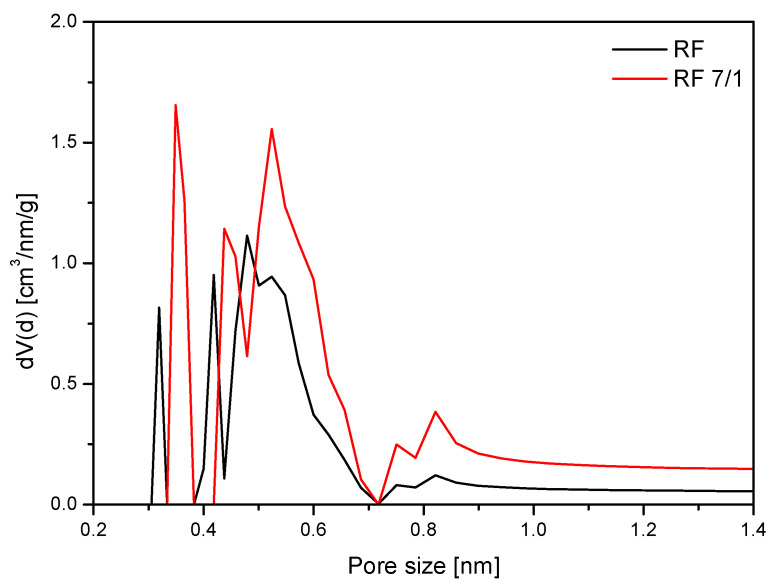
Comparison of the pore size distribution (PSD) of unmodified sample and sample modified with potassium oxalate and without heat treatment.

Figure 6 shows a comparison of the pore size distribution of unmodified sample RF and sample modified with potassium oxalate. The addition of potassium oxalate resulted in development of total pore volume from 0.25 to 0.49 cm^3^/g. Moreover, a noticeable increase in the proportion of pores of size 0.35 and 0.55 nm significantly improved CO_2_ uptake.

**Figure 7:**
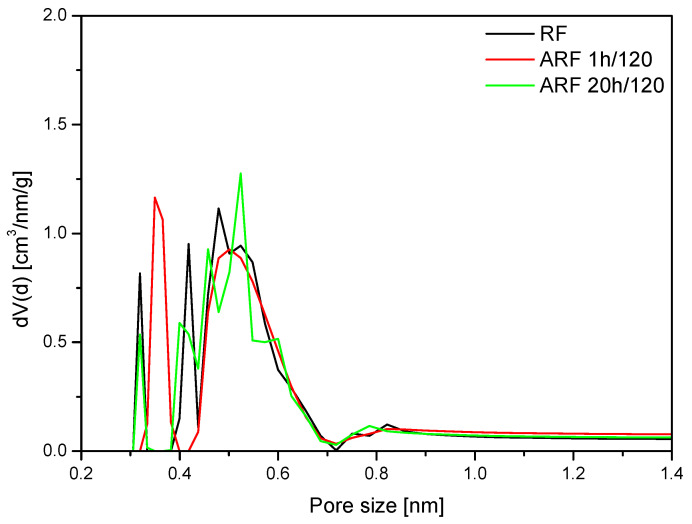
Pore size distributions of the unmodified samples.

Figure 7 shows the PSD of unmodified samples obtained at different conditions. Pore size distributions of all samples are similar. A high content of pores in the range 0.3–0.4 nm and 0.4–0.6 nm can be observed. Similar pore size distributions of the samples correspond to the similar CO_2_ adsorption values.

**Figure 8:**
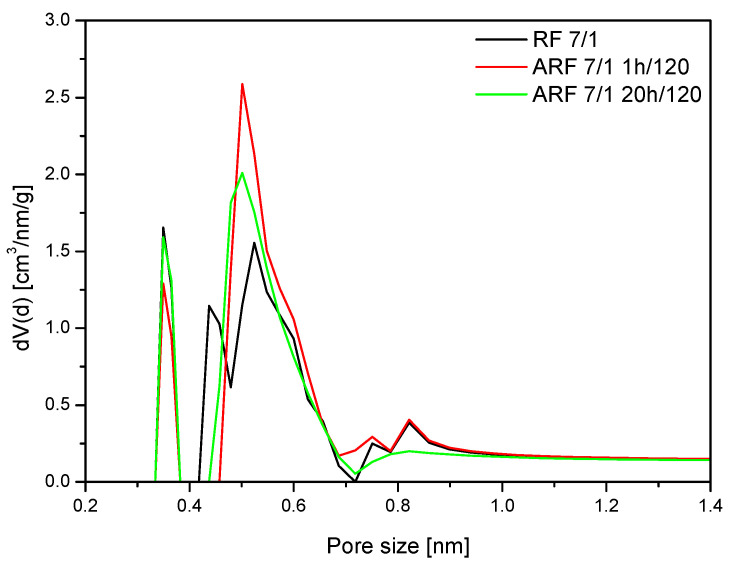
Pore size distributions of the samples modified with potassium oxalate.

Results of treatment in the autoclave are more noticeable for samples modified with potassium oxalate (Figure 8). After 1 h of heat treatment, an increase of the content of 0.35 nm pores and 0.55 nm pores can be observed. Interestingly, further heat treatment (20 h) did not improve porosity.

Thermally treated samples exhibited higher CO_2_ uptake values at 25 °C. Although the content of 0.35 nm pores for samples modified with potassium oxalate is similar, a higher content of 0.55 nm pores for the sample ARF_7/1_1 h/120 can be observed. This difference in pore structure is associated with higher CO_2_ adsorption at 25 °C.

Thermal treatment of the modified samples is necessary for the improvement of CO_2_ uptake at 25 °C.

Moreover, comparing the porosity of the unmodified and modified samples, the application of potassium oxalate resulted in a shift of the narrow pores below 0.4 nm towards higher size, and made these pores more effective for adsorption of CO_2_ molecules. On the other hand, the fraction of 0.55 nm pores also increased. Potassium ions likely broadened the smaller pores and activated them in the matter of CO_2_ adsorption.

The influence of heating on the porosity of samples is also visible in Figure 8. A sample thermally treated for 1 h exhibited almost 1 mmol/g higher CO_2_ uptake value at 25 °C than untreated sample, which correlates with differences between content of the 0.55 nm pores for these samples.

## 3. Materials Preparation

Sample preparation was performed as follows: First, 0.6 g of resorcinol was dissolved in an aqueous alcohol solution composed of 60 mL distilled water and 24 mL of ethanol. Next, to adjust pH, 0.3 mL of ammonium hydroxide (25 wt%) was added into the solution. To study the influence of the addition of potassium oxalate, 4.95 g of potassium oxalate was added, and the mixture stirred until the potassium oxalate was completely dissolved (the weight ratio of potassium/carbon was 7:1). Samples without potassium oxalate were also prepared. Regardless of whether the potassium oxalate was added or not, 0.9 mL of formaldehyde (37 wt%) was then added to the solution and mixed using a magnetic stirrer at ambient conditions to facilitate polycondensation reaction. After 24 h, the mixture was transferred into an autoclave BR-100 (Berghof, Eningen, Germany) designed and manufactured by Products + Instruments. The processes were conducted under different experimental conditions, e.g., temperature (120 and 200 °C) and reaction time (15 min and 1, 12, and 20 h). After treatment in the autoclave, the products were dried for 48 h at 80 °C and then carbonized in a high-temperature furnace (HST 12/400 Carbolite) (Carbolite, Derbyshire, UK) under argon atmosphere with the temperature increasing from 20 to 350 °C at a heating rate of 1 °C/min and holding time 2 h and from 350 to 700 °C at a heating rate of 1 °C/min. The carbonization temperature (700 °C) was chosen based on our previous research [10,18]. When a temperature of 700 °C was reached, carbonization continued for 2 h. Afterwards, the sample was cooled to room temperature under argon atmosphere. The final product was washed two times with 200 mL distilled water and dried for 48 h at 80 °C under atmosphere.

Samples without pressure treatment were also prepared. The experimental conditions were the same as described above, except samples which were directly calcined after the 24 h polycondensation reaction without pressure treatment. 

In this paper, materials were denoted as ARF for samples without addition of potassium oxalate or ARF_7/1 with addition of potassium oxalate, i.e., ARF_15 min/120, refers to the sample obtained without addition of potassium oxalate and the first number is the reaction time (15 min), the second the reaction temperature (120 °C), and the designation ARF_7/1_15 min/120 signifies the sample was obtained with addition of potassium salt with the same experimental conditions as for the previously mentioned sample (reaction time 15 min, reaction temperature 120 °C). The materials not treated in the autoclave with and without potassium oxalate were denoted as RF_7/1 and RF, respectively. 

## 4. Materials Characterization

Morphology of the samples was investigated using a ZEISS Scanning Electron Microscope (Carl Zeiss Microscopy GmbH, Jena, Germany) and FEI Tecnai F20 (Thermo Fisher Scientific, Hillsboro, OR, USA) for high-resolution transmission electron microscopy (HRTEM). The phase composition was investigated with X-ray diffraction (XRD) using Cu Kα radiation (λCu Kα = 0.1540 nm) on an Empyrean, Panalytical. Phase identification was performed using HighScore+ and the ICDD PDF-4+ 2015 database. Thermal stability of the materials was investigated using Thermal Gravimetric Analysis (TGA), using a STA 449 C thermobalance (Netzsch Holding, Selb, Germany). Approximately 10 mg of each sample was heated to 950 °C at 10 °C/min under air atmosphere.

Characterization of porosity was performed using N_2_ adsorption/desorption on a Quadrasorb™ automatic system (Quantachrome Instruments, Boynton Beach, FL, USA) at −196 °C. The Brunauer–Emmett–Teller (BET) equation was used to determine surface areas (S_BET_), and S_BET_ was determined in the relative pressure range of 0.05–0.2. The total pore volume, V_p_, was calculated from the volume of nitrogen held at the highest relative pressure (p/p0 = 0.99).

Before each adsorption experiment, samples were outgassed at 250 °C under a vacuum of 1 × 10^−5^ mbar for 12 h using a masterprep multi-zone flow/vacuum degasser from Quantachrome Instruments to remove adsorbed species that could intervene in the adsorption processes.

Carbon dioxide adsorption isotherms at 0 and 25 °C were measured using the same Quadrasorb™ automatic system (Quantachrome Instruments) with a relative pressure range between 0 and 0.98. Pore size distribution (PSD) of the samples was calculated from CO_2_ sorption isotherms at 0 °C using NLDFT model. 

## 5. Conclusions

Microporous carbon spheres with high CO_2_ adsorption efficiency were synthesized in an autoclave and also at pressureless conditions, and show that pressure treatment is not necessary to produce high-quality carbon spheres. The morphology and porosity of the spheres produced without activation step under pressureless (atmospheric) conditions were not significantly different from those obtained at higher pressures. CO_2_ uptake was also unaffected by elevated pressure synthesis.

The practical implication associated with these results is that the carbon spheres can be produced without pressure treatment, which enables scale upgrading of the production process, which is not limited by the volume of the pressure equipment.

The use of potassium oxalate as an activator had a significant effect on the morphology and carbon dioxide adsorption properties—far greater than the effect of applied pressure. Generally, a non-uniform size distribution of spherical particles was observed, but simultaneously higher values of surface area and total pore volumes were achieved. Treatment of the activated carbon materials in the autoclave significantly improved the CO_2_ uptake at 25 °C, but had no effect at 0 °C.

## Figures and Tables

**Table 1:** Physicochemical properties of the tested samples.

Designation of the Sample	S_BET_	Total Pore Volume	CO_2_ Adsorption at 0 °C	CO_2_ Adsorption at 25 °C
	(m^2^/g)	(cm^3^/g)	(mmol/g)	(mmol/g)
**The Samples without Addition of Potassium Oxalate**				
RF	472	0.25	3.59	2.52
ARF_15 min/120	476	0.25	3.61	2.46
ARF_15 min/200	478	0.26	3.53	2.49
ARF_1 h/120	474	0.26	3.78	2.52
ARF_1 h/200	483	0.27	3.73	2.52
ARF_12 h/120	470	0.26	3.60	2.46
ARF_20 h/120	486	0.28	3.59	2.44
ARF_12 h/200	462	0.25	3.70	2.52
ARF_20 h/200	473	0.29	3.51	2.55
**The Samples with Addition of Potassium Oxalate**				
RF_7/1	904	0.49	6.25	3.74
ARF_7/1_15 min/120	903	0.49	5.96	4.41
ARF_7/1_1 h/120	969	0.51	6.35	4.60
ARF_7/1_12 h/120	847	0.46	6.08	4.06
ARF_7/1_20 h/120	831	0.45	6.02	4.08
ARF_7/1_12 h/200	923	0.49	6.07	4.14
ARF_7/1_20 h/200	986	0.54	6.03	4.55

**Table 2:** Comparison of the CO_2_ adsorption values for potassium salt-activated carbon materials from different precursors at 1 bar.

Carbon Precursor	CO_2_ Adsorption at 0 °C	CO_2_ Adsorption at 25 °C	Reference
Resorcinol-formaldehyde resin	6.30	4.70	[24]
Coal tar pitch	6.00	4.03	[47]
Fern leaves	4.52	4.12	[49]
Carrot peels	5.64	4.18	[49]
Starch	4.40	3.40	[50]
Pinecone biochar	7.90		[8]
Waste coffee grounds	7.50	4.21	[51]

## References

[1] European Commission, Brussels, 11.12.2019 COM(2019) 640 Final. https://ec.europa.eu/info/sites/info/files/european-green-deal-communication_en.pdf.

[2] (2019). National Academies of Sciences, Engineering, and Medicine. Negative Emissions Technologies and Reliable Sequestration: A Research Agenda.

[3] (2018). European Academies’ Science Advisory Council. Negative Emission Technologies: What Role in Meeting Paris Agreement Targets?.

[4] Rifka T., Morosuk T., Tsatsaronis G. (2019). Carbon capture and storage using low-temperature post-combustion technologies. Energy Sources Part A.

[5] Gómez-Díaz D., Muñiz-Mouro A., Navaza J.M., Rumbo A. (2020). Diamine versus amines blend for CO_2_ chemical absorption. AIChE J..

[6] Abd A.A., Naji S.Z., Hashim A.S., Othman M.R. (2020). Carbon dioxide removal through physical adsorption using carbonaceous and non-carbonaceous adsorbents: A review. J. Environ. Chem. Eng..

[7] Rosli A., Ahmad A.L., Low S.C. (2020). Enhancing membrane hydrophobicity using silica end-capped with organosilicon for CO_2_ absorption in membrane contactor. Sep. Purif. Technol..

[8] Gadipelli S., Howard C.A., Guo J., Skipper N.T., Zhang H., Shearing P.R., Brett D.J.L. (2020). Superior multifunctional activity of nanoporous carbons with widely tunable porosity: Enhanced storage capacities for carbon-dioxide, hydrogen, water, and electric charge. Adv. Energy Mater..

[9] D’Alessandro D.M., Smit B., Long J.R. (2010). Carbon dioxide capture: Prospects for new materials. Angew. Chem. Int. Ed..

[10] Staciwa P., Narkiewicz U., Sibera D., Moszyński D., Wróbel R.J., Cormia R.D. (2019). Carbon spheres carbon spheres as CO_2_ sorbents. Appl. Sci..

[11] Khandaker T., Hossain M.S., Dhar P.K., Rahman S., Hossain A., Ahmed M.B. (2020). Efficacies of carbon-based adsorbents for carbon dioxide capture. Processes.

[12] Qiao W., Song Y., Lim S., Hong S., Yoon S., Mochida I., Imaoka T. (2006). Carbon nanospheres produced in an arc-discharge process. Carbon.

[13] Yang S., Zeng H., Zhao H., Zhang H., Cai W. (2011). Luminescent hollow carbon shells and fullerene-like carbon spheres produced by laser ablation with toluene. J. Mater. Chem..

[14] Choma J., Jamioła D., Augustynek K., Marszewski M., Gao M., Jaroniec M. (2012). New opportunities in Stöber synthesis: Preparation of microporous and mesoporous carbon spheres. J. Mater. Chem..

[15] Mi Y., Hu W., Dan Y., Liu Y. (2008). Synthesis of carbon micro-spheres by a glucose hydrothermal method. Mater. Lett..

[16] Yang W., Feng Y., Chu W. (2014). Comparative study of textural characteristics on methane adsorption for carbon spheres produced by CO_2_ Activation. Int. J. Chem. Eng..

[17] Rey-Raap N., Villanueva S.F., Menéndez J., Arenillas A. (2017). Microporous carbon spheres derived from resorcinol-formaldehyde solutions. A new approach to coat supports. Microporous Mesoporous Mater..

[18] Sibera D., Narkiewicz U., Kapica J., Serafin J., Michalkiewicz B., Wróbel R.J., Morawski A.W. (2018). Preparation and characterisation of carbon spheres for carbon dioxide capture. J. Porous Mater..

[19] Tian H., Liu J., O’Donnell K., Liu T., Liu X., Yan Z., Liu S., Jaroniec M. (2016). Revisiting the Stöber method: Design of nitrogen-doped porous carbon spheres from molecular precursors of different chemical structures. J. Colloid Interface Sci..

[20] Zhao J., Niu W., Zhang L., Cai H., Han M., Yuan Y., Majeed S., Anjum S., Xu G. (2012). A Template-free and surfactant-free method for high-yield synthesis of highly monodisperse 3-aminophenol–formaldehyde resin and carbon nano/microspheres. Macromolecules.

[21] Wickramaratne N.P., Jaroniec M. (2013). Activated carbon spheres for CO_2_ adsorption. ACS Appl. Mater. Interfaces.

[22] Liu J., Qiao S., Liu H., Chen J., Orpe A., Zhao D., Lu G.Q. (2011). (Max) Extension of the stöber method to the preparation of monodisperse resorcinol-formaldehyde resin polymer and carbon spheres. Angew. Chem. Int. Ed..

[23] Wickramaratne N.P., Xu J., Wang M., Zhu L., Dai L., Jaroniec M. (2014). Nitrogen enriched porous carbon spheres: Attractive materials for supercapacitor electrodes and CO_2_ adsorption. Chem. Mater..

[24] Ludwinowicz J., Jaroniec M. (2015). Potassium salt-assisted synthesis of highly microporous carbon spheres for CO2 adsorption. Carbon.

[25] Wang X., Zhou J., Xing W., Liu B., Zhang J., Lin H., Cui H., Zhuo S. (2017). Resorcinol–formaldehyde resin-based porous carbon spheres with high CO_2_ capture capacities. J. Energy Chem..

[26] Pari G., Darmawan S., Prihandoko B. (2014). Porous carbon spheres from hydrothermal carbonization and KOH activation on Cassava and tapioca flour raw material. Procedia Environ. Sci..

[27] Wickramaratne N.P., Jaroniec M. (2013). Importance of small micropores in CO_2_capture by phenolic resin-based activated carbon spheres. J. Mater. Chem. A.

[28] Okunev A., Sharonov V., Aristov Y., Parmon V. (2000). Sorption of carbon dioxide from wet gases by K_2_CO_3_-in-porous matrix: Influence of the matrix nature. React. Kinet. Catal. Lett..

[29] Tripathi N.K. (2018). Porous carbon spheres: Recent developments and applications. AIMS Mater. Sci..

[30] Wang Y., Chang B., Guan D., Cheng F. (2015). Mesoporous activated carbon spheres derived from resorcinol-formaldehyde resin with high performance for supercapacitors. J. Solid State Electrochem..

[31] Lee S.-Y., Park S.-J. (2013). Determination of the optimal pore size for improved CO_2_ adsorption in activated carbon fibers. J. Colloid Interface Sci..

[32] Casco M.E., Martínez-Escandell M., Silvestre-Albero J., Rodríguez-Reinoso F. (2014). Effect of the porous structure in carbon materials for CO_2_ capture at atmospheric and high-pressure. Carbon.

[33] Chen L., Watanabe T., Kanoh H., Hata K., Ohba T. (2017). Cooperative CO_2_ adsorption promotes high CO_2_ adsorption density over wide optimal nanopore range. Adsorpt. Sci. Technol..

[34] Staciwa P., Sibera D., Pełech I., Narkiewicz U., Łojkowski W., Dąbrowska S., Cormia R. Effect of microwave assisted solvothermal process parameters on carbon dioxide adsorption properties of microporous carbon materials. Micropor. Mesopor. Mater..

[35] Xu S., Liu C., Ye F., Guo Y., Wiezorek J.M. (2017). Alkali-assisted hydrothermal route to control submicron-sized nanoporous carbon spheres with uniform distribution. Colloids Surf. A Physicochem. Eng. Asp..

[36] Liu X., Song P., Hou J., Wang B., Xu F., Zhang X. (2018). Revealing the dynamic formation process and mechanism of hollow carbon spheres: From bowl to sphere. ACS Sustain. Chem. Eng..

[37] Kukulka W., Wenelska K., Baca M., Chen X., Mijowska E. (2018). From hollow to solid carbon spheres: Time-dependent facile synthesis. Nanomaterials.

[38] Juhl A.C., Schneider A., Ufer B., Brezesinski T., Janek J., Fröba M. (2016). Mesoporous hollow carbon spheres for lithium–sulfur batteries: Distribution of sulfur and electrochemical performance. Beilstein J. Nanotechnol..

[39] Krishnamurthy G., Namitha R. (2013). Synthesis of structurally novel carbon micro/nanospheres by low temperature-hydrothermal process. J. Chil. Chem. Soc..

[40] Lima A.M.F., Musumeci A.W., Liu H.-W., Waclawik E.R., Silva G.G. (2009). Purity evaluation and influence of carbon nanotube on carbon nanotube/graphite thermal stability. J. Therm. Anal. Calorim..

[41] Long J.W., Laskoski M., Keller T.M., Pettigrew K.A., Zimmerman T.N., Qadri S.B., Peterson G.W. (2010). Selective-combustion purification of bulk carbonaceous solids to produce graphitic nanostructures. Carbon.

[42] Datsyuk V., Kalyva M., Papagelis K., Parthenios J., Tasis D., Siokou A., Kallitsis I., Galiotis C. (2008). Chemical oxidation of multiwalled carbon nanotubes. Carbon.

[43] Sing K.S.W. (1982). Reporting physisorption data for gas/solid systems with special reference to the determination of surface area and porosity (Provisional). Pure Appl. Chem..

[44] Thommes M., Kaneko K., Neimark A.V., Olivier J.P., Rodriguez-Reinoso F., Rouquerol J., Sing K.S. (2015). Physisorption of gases, with special reference to the evaluation of surface area and pore size distribution (IUPAC Technical Report). Pure Appl. Chem..

[45] Musa M.S., Sanagi M.M., Nur H., Ibrahim W.A.W. (2015). Understanding pore formation and structural deformation in carbon spheres during KOH activation. Sains Malays..

[46] Heidarinejad Z., Dehghani M.H., Heidari M., Javedan G., Ali I., Sillanpää M. (2020). Methods for preparation and activation of activated carbon: A review. Environ. Chem. Lett..

[47] Liu J., Liu X., Sun Y., Sun C., Liu H., Stevens L.A., Li K., Snape C.P. (2018). High density and super ultra-microporous-activated carbon macrospheres with high volumetric capacity for CO_2_ capture. Adv. Sustain. Syst..

[48] Lendzion-Bieluń Z., Czekajło Ł., Sibera D., Moszyński D., Sreńscek-Nazzal J., Morawski A., Wrobel R.J., Michalkiewicz B., Arabczyk W., Narkiewicz U. (2017). Surface characteristics of KOH-treated commercial carbons applied for CO_2_ adsorption. Adsorpt. Sci. Technol..

[49] Serafin J., Narkiewicz U., Morawski A.W., Wróbel R.J., Michalkiewicz B. (2017). Highly microporous activated carbons from biomass for CO_2_ capture and effective micropores at different conditions. J. CO_2_ Util..

[50] Li Y., Li D., Rao Y., Zhao X., Wu M. (2016). Superior CO_2_, CH_4_, and H_2_ uptakes over ultrahigh-surface-area carbon spheres prepared from sustainable biomass-derived char by CO_2_ activation. Carbon.

[51] Travis W., Gadipelli S., Guo Z. (2015). Superior CO_2_ adsorption from waste coffee ground derived carbons. RSC Adv..

[52] Presser V., McDonough J.K., Yeon S.-H., Gogotsi Y. (2011). Effect of pore size on carbon dioxide sorption by carbide derived carbon. Energy Environ. Sci..

